# A qualitative study of how stigma influences HIV services for transgender men and women in Nigeria

**DOI:** 10.1002/jia2.25933

**Published:** 2022-07-12

**Authors:** Waimar Tun, Julie Pulerwitz, Elizabeth Shoyemi, Anita Fernandez, Adepeju Adeniran, Franklin Ejiogu, Olusegun Sangowawa, Krista Granger, Osasuyi Dirisu, Adebola A. Adedimeji

**Affiliations:** ^1^ Social and Behavioral Science Research Population Council Washington DC USA; ^2^ Centre for Population and Health Initiatives Lagos Nigeria; ^3^ Population Council Lagos Nigeria; ^4^ TIERS Lagos Nigeria; ^5^ Crème de la Crème Lagos Nigeria; ^6^ Population Council Abuja Nigeria; ^7^ Policy Innovation Unit Nigerian Economic Summit Group Lagos Nigeria

**Keywords:** gender‐affirming, HIV, HIV care continuum, stigma, transgender, transgender‐inclusive services

## Abstract

**Introduction:**

Transgender men and women in Nigeria experience many barriers in accessing HIV prevention and treatment services, particularly given the environment of transphobia (including harassment, violence and discrimination) and punitive laws in the country. HIV epidemic control in Nigeria requires improving access to and quality of HIV services for key populations at high risk, including transgender men and women. We assessed how stigma influences HIV services for transgender people in Lagos, Nigeria.

**Methods:**

In‐depth interviews (IDIs) and focus group discussions were conducted with transgender men (*n* = 13) and transgender women (*n* = 25); IDIs were conducted with community service organization (CSO) staff (*n* = 8) and healthcare providers from CSO clinics and public health facilities (*n* = 10) working with the transgender population in March 2021 in Lagos. Content analysis was used to identify how stigma influences transgender people's experiences with HIV services.

**Results and discussion:**

Three main findings emerged. First, gender identity disclosure is challenging due to anticipated stigma experienced by transgender persons and fear of legal repercussions. Fear of being turned in to authorities was a major barrier to disclose to providers in facilities not affiliated with a transgender‐inclusive clinic. Providers also reported difficulty in eliciting information about the client's gender identity. Second, respondents reported lack of sensitivity among providers about gender identity and conflation of transgender men with lesbian women and transgender women with being gay or men who have sex with men, the latter being more of a common occurrence. Transgender participants also reported feeling disrespected when providers were not sensitive to their pronoun of preference. Third, HIV services that are not transgender‐inclusive and gender‐affirming can reinforce stigma. Both transgender men and women spoke about experiencing stigma and being refused HIV services, especially in mainstream public health facilities, as opposed to transgender‐inclusive CSO clinics.

**Conclusions:**

This study highlights how stigma impedes access to appropriate HIV services for transgender men and women, which can have a negative impact along the HIV care continuum. There is a need for transgender‐inclusive HIV services and competency trainings for healthcare providers so that transgender clients can receive appropriate and gender‐affirming HIV services.

## 1 INTRODUCTION

In sub‐Saharan Africa (SSA), transgender persons experience a high burden of HIV and sexually transmitted infections (STIs) [1, 2, 3, 4, 5, 6, 7], physical and sexual violence [8, 9], stigma and discrimination [10, 11, 12], mental health issues [8, 12] and inadequate access to HIV prevention services [13]. In Nigeria, HIV prevalence data among transgender persons are sparse; HIV prevalence among men who have sex with men and transgender women (TGW) (combined) attending community clinics was reported to be 44–66% [14].

Multiple intersecting stigmas related to HIV status, sexual orientation, transphobia and homophobia also influence the experience of transgender people. Evidence of the negative impact of intersectional stigma on HIV risk‐reduction behaviours and antiretroviral therapy initiation and retention is growing [15, 16, 17, 18]. Transgender populations often navigate several challenges, including various forms of stigma and laws criminalizing same‐sex relationships [5, 10, 19, 20], when trying to access services for HIV prevention and care [11, 21, 22]. Nigeria's Same Sex Marriage Prohibition Act (2013) prohibits same‐sex relationships and organizations supporting people in such relationships, and creates a challenge to provision of and access to services to anyone believed to be engaging in same‐sex relationships, including transgender individuals [20, 23, 24].

In recent years, the number of programmes addressing the needs of transgender populations (primarily HIV services) implemented in SSA countries has increased [25, 26], including the provision of gender‐sensitive and stigma reduction trainings for healthcare providers [27, 28, 29]. As HIV programmes attempt to expand their services to the transgender population, there is a need to better understand how stigma influences existing services in specific contexts like Nigeria.

## 2 METHODS

In March 2021, in‐depth interviews (IDIs) and focus group discussions (FGDs) were conducted by trained interviewers/moderators in Lagos State with 25 TGW, 13 transgender men (TGM), 10 healthcare providers who work with transgender clients and 8 community service organization (CSO) representatives working with transgender clients. Table 1 describes the study population and methodology. CSO representatives provided input into all study guides. TGW/TGM guides were pilot tested. Participants provided written informed consent. Some CSO (*n* = 4) and provider (*n* = 1) interviews were conducted by phone; their consent was obtained over the phone and audio‐recorded. All interviews were audio‐recorded; a notetaker took notes of FGD sessions. All interviews were translated/transcribed. The study coordinator reviewed all transcriptions for accuracy. Thematic content analysis was used to analyse the data using NVivo 12 software to identify stigma‐related gaps in HIV service delivery. Four researchers (including one co‐investigator) with a good understanding of local context reviewed the data and developed the codebook through consensus. Coding was deductive and *a priori* codes guided the process. These were discussed by the research team through an iterative process. Team meetings were used to resolve disagreement in coding among coders. The investigative team discussed and categorized the codes into three main themes of: (1) challenges with gender identity disclosure to providers; (2) lack of sensitivity among providers about gender identity; and (3) HIV services not being transgender‐inclusive and gender‐affirming. Data saturation was achieved. The study was approved by the Population Council's Institutional Review Board and the Nigerian Institute of Medical Research.

**Table 1:** Study population, methodology and sample characteristics

	Transgender women (*N* = 25)	Transgender men (*N* = 13)	Transgender‐inclusive CSO staff (*N* = 8)	Healthcare providers (*N* = 10)
Type of interviews	10 IDIs 3 FGDs (5 participants per FGD) (in‐person)	8 IDIs 1 FGD (5 participants per FGD) (in‐person)	8 IDIs (by phone or in‐person)	10 IDIs (by phone or in‐person)
Eligibility criteria	Self‐identify as transgender or have discordant responses to the two‐step questions on sex assigned at birth and gender identity; 18 years or older		Having worked at least 6 months for a CSO working with transgender population and being in a position that interacts with transgender community or makes decisions for transgender programmes; 18 years or older	Having worked at least 6 months at one of the transgender‐inclusive clinics or public health facilities that have been trained to provide transgender‐inclusive services through an Elton John AIDS Foundation‐funded programme
Sampling and recruitment	Five transgender‐inclusive CSOs used convenient sampling and selected transgender men and women (diversified by age) who use their programmes and services; Participants also referred peers. All participants referred by the CSOs agreed to participate.		Purposively sampled from among five transgender‐inclusive CSOs in Lagos; selected participants who had ample knowledge of services for transgender men and women	Convenience sample
Place of interview	At transgender‐inclusive community, health clinic operated by CSO serving high‐risk key populations		Phone interview or at CSO office	Phone interview or at place of work (health facility/clinic)
Duration	IDIs: 1 hour FGDs: 1.5 hours		IDIs: 1 hour	IDIs: 1 hour
Topics of inquiry	HIV and sexual health needs of TGM/TGW and challenges in accessing services Experiences accessing HIV and sexual health services HIV/STI risk and vulnerabilities of TGM/TGW Experiences of stigma and discrimination		HIV/STI risk and vulnerabilities of TGM/TGW HIV and sexual health needs of TGM/TGW and challenges in accessing services Strategies to improve HIV and sexual health services for TGM/TGW	Attitudes towards TGM/TGW and providing services to TGM/TGW HIV and sexual health needs of TGM/TGW and challenges in accessing services Challenges in providing services for TGM/TGW
Interviewer	Self‐identified cisgender man (*n* = 3) Self‐identified cisgender woman (*n* = 2) [Both men and women interviewed TGM and TGW]		Self‐identified cisgender male (*n* = 3) Self‐identified cisgender female (*n* = 2)	Self‐identified cisgender male (*n* = 3) Self‐identified cisgender female (*n* = 2)
Language of interview	Mix of English and Pidgin English		English (as preferred by participants)	English (as preferred by participants)
Reimbursement	5000 Nairas (US$ 12)		5000 Nairas (US$ 12)	None
Cadre of staff			Director (*n* = 4) Programme Officer/Other (*n* = 3) Community mobilizer (*n* = 1)	Doctor (*n* = 4) Counsellor (*n* = 4) Nurse (*n* = 2)
Health facility type				CSO clinics (*n* = 4) Public health facilities (*n* = 6)
Median age (IQR)	24 (22, 27)	27 (25, 31)		
HIV status	2 HIV positive	All HIV negative or unknown		

Abbreviations: CSO, community service organization; FGD, focus group discussion; IDI, in‐depth interview; IQR, inter‐quartile range; TGM, transgender men; TGW, transgender women.

## 3 RESULTS AND DISCUSSION

Three main themes emerged: (1) challenges with gender identity disclosure to providers; (2) lack of sensitivity among providers about gender identity; and (3) HIV services not being transgender‐inclusive and gender‐affirming.

**1. *Gender identity disclosure is challenging due to anticipated stigma experienced by transgender persons and fear of legal repercussions***

Disclosure of ones’ gender identity can theoretically help facilitate more tailored HIV prevention services, as sensitized providers could then have a better understanding of the potential needs and vulnerabilities of clients. However, such disclosure to providers remains a great challenge. For example, anticipation of legal repercussions may impede transgender clients from disclosing due to fear of being turned in to authorities if their gender identity was exposed.

*Like in Nigeria now, we have laws against them. So for them to even come out even if you are telling them we are friendly, we are this, they will still be scared of “I hope you won't report them to ‘you know’”*. (***Provider, Public Health Facility [PHF]***)

*You're not encouraged to go [to hospitals]. Because it is not legalized, one. Two, it is not officially known. And you might be seen as a strange person when you start voicing out your problems*. ***(TGW, 21, IDI)***

Some providers reported difficulty in eliciting information about the client's gender identity, even providers who had received transgender competency training and intended to discuss this topic to help meet the clients’ needs. Other providers reportedly felt uncomfortable with the topic in general.

*Sometimes it gets uncomfortable because even the client that is transgender or MSM, they themselves are not comfortable. …They might not really want to come out. (**Provider, PHF)***

*A lot of health workers still find it so uncomfortable; you know to treat you, to identify with you as maybe a MSM or transgender. …as you present to them your sexual orientation, they still want to ignore it*. ***(Provider, PHF)***

Because stigma inhibits disclosure of gender identity, it can restrict access to appropriate HIV prevention and care services, as has been reported in related studies in other contexts [30, 31, 32, 33]. Relatedly, providers from facilities that have received gender/sexual diversity training indicated that although their facilities offer sensitized HIV services, they are still not well equipped to offer other services that transgender clients need. For example, they refer to transgender‐inclusive CSO clinics for certain STIs (e.g. anogenital warts) and mental health counselling.

*I refer [transgender clients] to [CSO clinic] where there is mental health services. …I would transfer the person where the person will go and get service and come back to continue his medication. **(Provider, PHF)***

**2. *Lack of sensitivity about gender identity and conflation of transgender men and women with being lesbian or gay/men who have sex with men (MSM)***

Both TGM and TGW spoke about providers not being sensitive to their requested pronoun and that they felt disrespected.

*I just feel it's still more of the discrimination. Some people not respecting your pronouns. When you tell them (providers), …when they call you Miss and you are like, no, I prefer to be called Mr, and they look at you like, “ah ah, as you are like this endowed with breast, which one is Mr?” They don't understand you. **(TGM, 26, IDI)***

The conflation of TGM with lesbian women and TGW with being gay or MSM was raised frequently even though they were not asked about these different groups. This conflation occurred naturally, even among providers who had received gender/sexual diversity training. This conflation appeared to be more common for TGW than TGM. Some providers assumed that MSM and TGW had similar health needs because they assumed both groups engage in anal sex.

*…this thing, gay, MSM, transgender …it's still the same thing. …Even though some goes to the extent of changing their reproductive systems …so I think apart from the physical structure, it's the same need because …it's still anal sex all of them do. **(Provider, PHF)***

*They [providers] do not understand the term “trans”, so they just stick to the word, “homo” and “gay”. **(TGW, 27, IDI)***

Studies that have documented the impact of widespread conflation of gender identity with sexual orientation [34, 35, 36, 37] highlight how this can limit access to and the effectiveness of HIV prevention services and the need to acknowledge transgender identities and address the unique needs of this population [36]. Provider awareness of, and respect for individual gender identity is critical for optimal delivery of HIV and other health services for Nigerian TGM and TGW [37]. Studies suggest that interventions that increase provider competence in delivering gender‐affirming services can facilitate transgender engagement in, as well as retention in, HIV care [38, 39] and can increase patient–provider trust and foster positive interactions [38, 40].

**3. *Offering HIV services without tailoring to transgender community needs can reinforce stigma***

Both TGM and TGW reported experiencing stigma related to their gender identity when accessing HIV services. CSO representatives and providers also spoke about the stigmatizing attitudes among HIV providers have towards transgender clients and occasions of provider refusal to attend to transgender clients. This is an example of intersectional stigma, where transgender individuals experience multiple stigmas (i.e. related to gender identity, as well as HIV—resulting in not receiving needed HIV services).

*That was the first and last time I went to the government hospital to get my HIV test and when the lady saw me, she now ask me for my sex,…I opened up to her that I was a transwoman, she was now saying …it's sinful you are going to hell, that HIV is connected to hell…I was like I wanted to leave that place immediately. **(TGW, 22, FGD)***

*… there was a time I went to get tested for HIV, it was at a public sector…the person was very rude to me on the basis that I was effeminate, and I still couldn't get the test done. Because after being insultive, she still told me that I should go. **(TGW, 24, IDI)***

These findings show that stigma acts as a barrier to HIV testing, and as a consequence, transgender people do not have the opportunity to even enter the HIV care continuum. This finding is consistent with other studies that have reported poor care continuum outcomes among transgender populations and that stigma and discrimination is one of the key impediments to better outcomes [38, 39, 41, 42, 43, 44, 45, 46, 47].

Transgender respondents and some providers also pointed to a distinction in the experience of stigma and discrimination depending on the type of facility where they accessed HIV services, with negative experiences reported at “mainstream” health facilities and more positive experiences reported at transgender‐inclusive CSO clinics.

*I don't go to normal general healthcare providers, but when it comes to queer health care providers, they treat me well but when it comes to the normal general local health care providers, they treat me very very bad which I don't even try to go there anymore. **(TGW, 23, IDI)***

*The healthcare providers [in ‘mainstream’ facilities] are not even knowledgeable enough to know that this people exist or even those who knows that they exist are still afraid …that if they provide services to these people, they may face the law. **(Provider, CSO clinic)***

Transgender clients felt uncomfortable about receiving physical examinations from providers, particularly in mainstream health facilities. Because of anticipated stigma, many transgender individuals are unwilling to access services from providers in mainstream facilities because they feel uncomfortable and disrespected by provider's insensitivity to gender identity and/or specific healthcare needs.

*Some transwomen are very shy of going to the hospital to meet a doctor and ask doctor I want to check if my ass is okay. **(TGW, 21, IDI)***

Most providers from the participating public health facilities (i.e. “mainstream”), however, appeared to not be aware of these challenges.

*If they come here, we'll take them as patients. We treat them normal; we don't look at the, the bad aspect of it in society, or what the law implies, we don't practice that kind of law here, we just take patients as patients. …I told them that ehm your practice is not a sin here. **(Provider, PHF)***

These findings highlight the varying experiences of stigma experienced by TGM and TGW in different healthcare settings, which seemed to depend on whether the facility was a transgender‐inclusive CSO facility and/or providers in the health facility had undergone transgender competency training. Suggestions for future interventions include a recommendation for providers in “mainstream” public facilities to undergo competency training in gender‐affirming services, particularly in HIV and STI units, to improve clinical practice. Trainings can include, for example, sessions on non‐stigmatizing and respectful care to gain skills to sensitively probe about gender identity and sexual behaviours so that appropriate services can be offered, correctly acknowledge clients gender identity and pronouns, and confidently conduct physical examinations for transgender clients. Moreover, having a focal person who can attend to transgender clients or having transgender peers or key opinion leaders [48] as navigators or case managers to facilitate linkage to services and navigate the appointment can help in creating a safe, enabling environment. Another stigma reduction strategy could be to coordinate more closely with CSOs that already offer gender‐affirming services to bring lessons learned to other facility settings.

Turning to study limitations, since the qualitative data were obtained from TGM and TGW who are connected to transgender‐inclusive CSOs, the views of those not connected to such CSOs may not be represented. Nevertheless, the findings are consistent with those reported in other settings but more importantly highlight the specific ways in which stigma creates gaps in the provision of and access to services for transgender people in Nigeria. A major strength is that this is one of the few studies about the effects of stigma on HIV services for the transgender population, including their own voices.

## 4 CONCLUSIONS

This novel study found that stigma impedes access to appropriate HIV services (such as HIV testing) for the Nigerian transgender population, a community that is highly marginalized and hidden in the Nigerian context. These findings call attention to the need to address clinical practice, and programmatic and policy gaps in availability and access to gender‐affirming HIV services for Nigerian transgender persons, especially within healthcare settings. There is also a need for the national HIV and STI service delivery guidelines to include specific language around transgender‐inclusive and gender‐affirming services. The current guidelines (2020) do not specifically include transgender persons [49]. Additional research to explore and pilot effective stigma reduction and competency skills‐building interventions focused on the transgender population could improve services along the HIV care continuum and ultimately HIV prevention and treatment outcomes in this population.

## COMPETING INTERESTS

All authors have no competing interests to report.

## AUTHORS’ CONTRIBUTIONS

WT, AAA and JP conceptualized, wrote and revised the manuscript. KG conducted literature review and contributed to the manuscript writing. AF oversaw the implementation of the data collection, assisted with data analysis and contributed to the manuscript writing. WT wrote the protocol for the study. OD trained data collectors and conducted the analysis. OS trained data collectors and helped with manuscript revision. ES assisted with data collection implementation, provided feedback on study tools and revision of the manuscript. AA and FE assisted with study tool reviews, data collection implementation and revision of the manuscript. All authors have read and approved the final manuscript.

## FUNDING

The study was funded by the Elton John AIDS Foundation.

## Data Availability

The data that support the findings of this study are available on request from the corresponding author.
